# A Comprehensive and Ultrasensitive Isotope Calibration Method for Soil Amino Compounds Using Orbitrap Mass Spectrometry

**DOI:** 10.1021/acs.analchem.5c01358

**Published:** 2025-06-12

**Authors:** Tao Li, Yuhua Li, Erika Salas, Ye Tian, Xiaofei Liu, Wolfgang Wanek

**Affiliations:** a Division of Terrestrial Ecosystem Research, Department of Microbiology and Ecosystem Science, Centre for Microbiology and Environmental Systems Science, 560173 University of Vienna, Djerassiplatz 1, A-1030 Vienna, Austria; b Doctoral School in Microbiology and Environmental Science, University of Vienna, Djerassiplatz 1, A-1030 Vienna, Austria; c Department of Soil and Environment, Swedish University of Agricultural Sciences, Lennart Hjelms väg 9, 756 51 Uppsala, Sweden; d State Key Laboratory for Subtropical Mountain Ecology of the Ministry of Science and Technology and Fujian Province, Fujian Normal University, Fuzhou 350007, China

## Abstract

Bound amino compounds (amino acid and amino sugar polymers) comprise a significant proportion (∼40%) of soil organic nitrogen and therefore represent an essential source of nitrogen for plant and microbial nutrition. The analysis of their content and isotope enrichment still represents a significant challenge due to the low isotope enrichment levels reached under near-native soil conditions and the lack of isotopically labeled standards for some key amino compounds. In this study, we used both a ^13^C-labeled and an unlabeled amino acid mixture to establish isotope calibration curves for 16 amino compounds, using the 6-aminoquinolyl-*N*-hydroxysccinimidyl carbamate (AQC) derivatization method and ultrahigh-performance liquid chromatography with high-resolution Orbitrap mass spectrometry (UPLC-Orbitrap MS). Molecular ions of AQC-derivatives for all standard amino compounds were identified at the expected *m*/*z* values of the respective isotopologues. The isotope calibration curves exhibited excellent linear fits across the whole ^13^C enrichment range and polynomial fits in the low ^13^C enrichment range (*R* ^2^ > 0.990). However, the polynomial fitting terms differed between individual amino acids. Subsequently, we developed equations to relate the calibrated regression terms to the physicochemical properties of the respective amino acids, here mainly the ratio of amino compound-C atoms to total C atoms in AQC-amino compound derivatives. Based on these regressions, we could ultimately predict isotope calibration curves for those amino compounds unavailable as ^13^C labeled standards, for example, muramic acid, hydroxyproline, and diaminopimelic acid. To test the model accuracy, we compared the outcomes of measured calibrations with predicted calibrations for amino acids where we had isotopically enriched standards. The results of linear regression between measured and predicted data were excellent, where *R* ^2^ was >0.97, and mean absolute (percentage) deviations, MAD and MAPD, were 0.334 and 15.8%. Finally, we applied both standard and predicted calibration curves to low ^13^C amended soil samples and unlabeled controls to test the applicability of our model. The limit of detection (LOD) as the minimum detectable atom % ^13^C incorporation of amino compounds ranged from 0.0003 to 0.14 atom % among different amino compounds. This general predictive model can be used to comprehensively quantify isotope enrichments across the entire soil amino compound profile, including amino sugars and proteinogenic and nonproteinogenic amino acids, providing valuable insights for a better understanding of the overall fate of different amino compounds in soils and other complex environmental systems.

## Graphical abstract

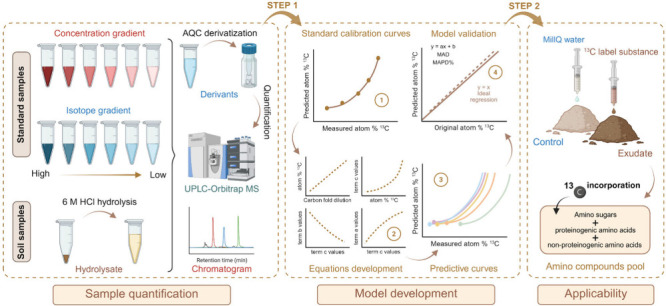

## Introduction

Soil amino compounds, such as amino acids and amino sugars, are crucial components of the soil organic nitrogen (N) pool.^1^ Amino sugars, including glucosamine (GlcN) and muramic acid (MurA), contribute approximately 5–8% of total N in soil, while amino acids contribute significantly more, accounting for about 20–50%.^2,3^ They play critical roles in soil N cycling in terrestrial ecosystems; in their free form amino compounds serve as essential N sources for soil microbes, and can also directly be utilized by some plants.^4,5^ Amino compounds predominantly exist as high molecular weight polymers in soils, such as amino sugars in peptidoglycan and chitin, and amino acids in proteins.^6^ Additionally, nonproteinogenic amino acids in soils are garnering increasing attention. Numerous studies have highlighted their significant roles in the ecological and physiological processes of soil-plant-microbe interactions, including compounds such as hydroxyproline (Hyp), gamma-aminobutyric acid (GAB), and 2,6-diaminopimelic acid (DAP).^7,8^ Aside of the conventional use of the amino sugars MurA and GlcN as bacterial and fungal necromass biomarkers, other amino compounds may allow the development of further biomarker approaches. Hydroxyproline might be applicable as proxy for plant necromass^9,10^ and different isomers of diaminopimelic acid in peptidoglycan may serve as bacterial necromass biomarker.^11^ The conventional acid hydrolysis method has been widely used to release polymeric amino compounds into free forms with high recovery and no isotopic fractionation. However, there are challenges in recovering all monomeric compounds from complex samples, particularly with amino acids.^12,13^ For instance, tryptophan (Trp) will be completely degraded under acidic condition, sulfur-containing amino acids will be oxidized, and asparagine (Asn) and glutamine (Gln) will be deamidated to aspartic acid (Asp) and glutamic acid (Glu), respectively.^12,13^ On the other hand, due to the low isotope enrichment levels reached under near-native soil conditions and the commercial unavailability or extremely high costs of isotopically labeled standards for some key amino compounds (e.g., MurA, DAP, Hyp),^6,14^ the comprehensive analysis of amino compound content and isotope enrichment in soil remains a significant challenge.

Stable isotope tracing, which employs isotopic tracers such as ^13^C or ^15^N to trace the dynamics of soil amino compounds, has emerged as a reliable tool in soil metabolomics and fluxomics research.^6,15^ Low isotope enrichments of amino compounds are commonly analyzed by gas chromatography (GC)-coupled compound-specific isotope ratio mass spectrometry (IRMS), for which currently no protocols are available to analyze amino acids and amino sugars in the same run, and therefore separate protocols are needed.^16,17^ Recently, a novel method coupling ultrahigh-performance liquid chromatography with high-resolution Orbitrap mass spectrometry (UPLC-Orbitrap MS) has been successfully implemented for the concentration and isotopic flux analysis of amino compounds.^6,18^ This allowed us to avoid the need for different derivatization steps for various nonvolatile amino compounds including amino acids and amino sugars, thereby reducing variability and saving time compared to GC-MS and GC-IRMS.^15,19^ The electrospray ionization (ESI)-Orbitrap Q Exactive MS system gently ionizes nonvolatile compounds in a high-voltage electric field and separates these ions by their mass-to-charge ratio based on their unique oscillation frequencies around a central electrode, enabling precise mass analysis at ultrahigh resolution and precision.^20,21^ Mass fragmentation analysis and high mass resolution (>70,000) allow high specificity of amino compound quantification and full separation of different isotopologues of amino compounds, e.g. differing by just one neutron deriving from ^13^C or ^15^N.^22,23^

Precolumn derivatization with 6-aminoquinolyl-*N*-hydroxysuccinimidyl carbamate (AQC), also available as AccQ-Tag Ultra Derivatization Kit by Waters, has been widely used in soil amino acid analysis to enhance separation and detection sensitivity.^24^ This method is favored for its straightforward derivatization process, strong fluorescent intensity, and stability of the resulting derivatives.^24,25^ AQC reacts with both primary and secondary amines, including amino acids, converting them into stable fluorescent derivatives with minimal interference from the major fluorescent byproduct, 6-aminoquinoline (AMQ).^25^ Other studies have also reported the successful application of AQC in the analysis of amino sugars, indicating that amino sugars can react with AQC to form stable derivatives suitable for separation and identification.^26–28^ However, AQC-derivatization introduces specific molecular fragments from AQC (*m*/*z* = 171.055) to the amino compound molecules, creating distinct amino compound-AQC derivatives.^29^ Therefore, isotope calibration of the derivatized molecules is imperative to accurately evaluate the true atom percentage of isotopes in native soil amino compounds.^30,31^

In this study, we applied both carbon-13 (^13^C)-labeled and unlabeled amino acid mixtures to establish standard calibration curves for various amino acids, using the AQC derivatization method and the UPLC-Orbitrap MS platform. Molecular ions of AQC-derivatives for all amino acids were identified at the expected *m*/*z* values of the respective isotopologues. Subsequently, we developed equations to relate the calibrated regression terms of standard curves to the physicochemical properties of the respective amino acids, such as molecular weight, C:N ratio or C dilution through added AQC-C atoms. Based on these equations, we could ultimately develop isotope calibration curves for those amino compounds unavailable as ^13^C labeled standards (e.g., MurA, Hyp, GAB, and DAP). Then, we evaluated the accuracy of the predicted isotope calibration model by performing linear regression between the original and predicted value. Furthermore, we assessed the applicability of this method on soil samples collected from a ^13^C labeling experiment in a mountain forest in Austria. To our knowledge, this is the first time to showcase the possibility and the application of a generalized isotope calibration method to the entire profile of amino compounds. This allows for comprehensive and highly sensitive quantification (subatom % ^13^C level) of the actual concentration and atom % ^13^C isotope enrichment in soil and other complex environmental systems, covering sources of isotopically labeled materials, calibration procedures for high and low enrichments, discussion of the mechanisms of deviations from linearity, and predicting the calibration parameters for compounds unavailable as isotopically enriched variants.

## Experimental Section

### Standards and Reagents

For concentration calibration, unlabeled single amino compounds, [including alpha-Alanine (α-Ala), beta-Alanine (β-Ala), Arginine (Arg), Aspartic acid (Asp), *meso*-2,6-diaminopimelic (mDAP), LL-2,6-diaminopimelic (LLDAP), gamma-Aminobutyric acid (GAB), Glutamic acid (Glu), Glycine (Gly), Histidine (His), Homoserine (Hse), Hydroxyproline (Hyp), Isoleucine (Ile), Leucine (Leu), Lysine (Lys), Methionine (Met), Phenylalaninie (Phe), Proline (Pro), Serine (Ser), Threonine (Thr), Tyrosine (Tyr), Valine (Val), Glucosamine (GlcN), Galactosamine (GalN), Mannosamine (ManN), and Muramic acid (MurA)] were purchased from Sigma-Aldrich (St. Louis, MO). For isotope calibration, a U–^13^C (97–99 atom %) labeled and an unlabeled algal amino acid mixture (extracted from a blue-green algal source comprising the 16 amino acids Ala, Arg, Asp, Glu, Gly, His, Ile, Leu, Lys, Met, Phe, Pro, Ser, Thr, Tyr, and Val) were purchased from Cambridge Isotope Laboratories (Tewksbury, MA). Methanol, hydrochloric acid, formic acid, and acetonitrile solution were also purchased from Sigma-Aldrich (LC-MS grade; St. Louis, MO). AccQ-Tag Ultra Derivatization Kit was obtained from Waters Corporation (Milford, MA, USA).

### Preparation of Calibration Standards and Derivatization

For concentration quantification, stock solutions of all single amino compound standards were prepared at 20 mM and mixed to give two combined standards (1 mM for each amino compound) to better separate isobaric amino acids, such as Ile/Leu, α-Ala/β-Ala, and mDAP/LLDAP. Combined standard 1 included Leu, β-Ala, LLDAP, Hyp, MurA, GlcN, GAB, and Hse, while combined standard 2 included the remaining amino acids. The concentrations of these two combined standards ranged from 300 μM to 2.344 μM by serial dilution. For isotope calibration, U–^13^C (97–99 atom %) labeled and unlabeled algal amino acid mixtures were respectively prepared into solutions of equal molar concentration. Then, we mixed them in an isotopic dilution series ranging from 98 atom % ^13^C to natural ^13^C abundance (∼1.1 atom % ^13^C). Each isotopic dilution was set up in triplicate for the five lowest ^13^C enrichment standards to allow the determination of the limits of detection (LOD) and limits of quantification (LOQ) of ^13^C enrichment in the amino compound-AQC derivatives.

The AccQ-Tag Ultra Derivatization Kit (Waters Corporation, Milford, MA, USA) was used to perform the derivatization reaction for both standards and samples, following the manufacturer’s protocol. The reaction was carried out using 70 μL AccQ-Tag Ultra borate buffer, 10 μL standards or samples, and 20 μL reconstituted AccQ-Tag Ultra AQC reagent. The solutions were thoroughly vortexed, left for 1 min at room temperature and then incubated in a heating block at 55 °C for 10 min. The solutions were then injected into the UPLC-Orbitrap MS system.

### Soil Sample Preparation

We used sieved (2 mm) soil samples from Achenkirch forest, Austria, to test the applicability of the method. The soil properties and site description can be found in a previous study.^32^ Low amounts of ^13^C-labeled synthetic root exudates (1.2 mg C mesocosm^–1^) were injected into control soils in *in situ* mesocosms (67 ± 5 g soil d.w. mesocosm^–1^). To mimic root exudates, a cocktail of three organic acids (citric acid, sodium acetate, and oxalic acid), two sugars (glucose and fructose) and 18 amino acids (Cambridge Isotope Laboratories) comprising 60%, 35% and 5% of the exudate C input respectively, was prepared at 10 atom % ^13^C enrichment and injected as solution.^33,34^ Soil samples were collected after 5 days from the whole mesocosms, sieved and air-dried. Aliquots of air-dried soil (0.04 g) were mixed with 10 mL 6 M HCl and heated at 105 °C for 8 h. After cooling to room temperature, the hydrolysates were filtered through cellulose acetate filter membranes (Sartorius, Goettingen, Germany) into 20 mL scintillation vials and dried with a nitrogen stream. The dried extracts were redissolved in 12 mL Milli-Q water, and the pH adjusted to 6.6–6.8 with 0.6 M KOH. The extracts were centrifuged (1600 *g*, 15 min) to remove iron precipitates, and the supernatant was freeze-dried. The freeze-dried extracts were dissolved in 8 mL methanol and centrifuged (1600 *g*, 10 min) to remove salt precipitates. The supernatant was then transferred and dried under a nitrogen stream. Finally, the dried extracts were redissolved in 1 mL Milli-Q water for AQC derivatization.

### UPLC-Orbitrap MS Instrumentation

Derivatized samples were analyzed using an Ultimate 3000 UPLC system (Thermo Fisher Scientific, Bremen, Germany) coupled to an Orbitrap Q Exactive HRMS system (Thermo Fisher Scientific) with heated ESI source. The system was operated in full-mass scan mode (*m*/*z* 150–1000) in positive ESI mode, as referenced in a previous study.^6^ Automatic gain control (AGC) target values were set to 3 × 10^6^. The necessary mass resolution to separate isotopologues increases with molecular mass, and derivatization increases the mass of the amino compounds by AQC addition. Following the mass resolution equation, it is possible to calculate the minimum mass spectrometric resolution required to resolve two closely spaced peaks, such as the ^13^C_1_- and ^15^N_1_-isotopologues.

1 $$R=\frac{m}{\Delta m}$$

with *R* being the required (minimum) resolution, *m* the *m*/*z* of the ion of interest (nominal mass of the compound), and *Δm* the smallest difference to resolve (here 0.0063 Da between ^13^C and ^15^N isotopologues).^35^ The derivatized AQC-molecules range between 250 and 450 *m*/*z* (Table S1) in monoisotopic mass, necessitating mass resolutions between 39,680 and 71,430. Therefore, the resolution was set to 70,000 to allow separation of ^13^C and ^15^N isotopologues.^6^ Other system parameters were as follows: spray voltage (3.5 kV), capillary temperature (300 °C), sheath gas (35 arbitrary units), and auxiliary gas (15 arbitrary units).

Amino compound-AQC derivatives were separated using a Waters AccQ-Tag Ultra C18 column (2.1 mm × 100 mm, 1.7 μm particles) with a preparative guard column (2.1 mm, 0.2 μm) (Milford, MA, USA). The column temperature was 55 °C. The separation was conducted using eluent A (Milli-Q water, 0.1% v/v formic acid) and eluent B (acetonitrile (ACN), 0.1% v/v formic acid) with the following gradient: 0–0.5 min, 0.1% B; 0.5–2.5 min, increase to 5% B; 2.5–8 min, increase to 20% B; 8–8.25 min, increase to 90% B; 8.25–11 min, constant at 90% B; and 11–11.2 min, decrease to 0.1% B for column re-equilibration. The injection volume was set to 1 μL, and the eluent flow rate was 0.4 mL min^–1^.

### Data Analysis

All samples and standards were processed based on their mass spectrometric signals using FreeStyle 1.7 (Thermo Scientific) and Skyline 23.1 (University of Washington, Seattle, WA) software. Concentrations of samples were determined via calibration by concentration standards, depicting the relationship between known concentration and peak area all as the sum of the peak areas of all isotopologues. By using the ratio of the analyte signal to the background noise, with the signal-to-noise ratio (S/N) set to 3 and 10,^36^ respectively, the concentrations of LOD (LOD_concentration_) and LOQ (LOQ_concentration_) of each amino compound were extrapolated from the lowest detected concentration within the linear range.^37^ The equations are as follows:

2 $${\mathrm{LOD}}_{\mathrm{concentration}}=\frac{\mathrm{lowest detected concentration}\times 3}{S/N_{\mathrm{lowest detected concentration}}}$$

3 $${\mathrm{LOQ}}_{\mathrm{concentration}}=\frac{\mathrm{lowest detected concentration}\times 10}{S/N_{\mathrm{lowest detected concentration}}}$$

Isotope enrichment of samples was calculated based on isotope calibration standards. For each amino compound, the atom % ^13^C was calculated from all measured ^13^C-related isotopologues using the relative abundance of their signal (*S*_*k*_) weighted by the number of isotopically labeled carbons (*k*) as follows:

4 atom %⁡C13=∑k=1n(k×Sk)/(n×∑k=0nSk)

where *n* is the total number of carbons in the amino compound molecule without considering AQC-addition of C atoms.^18,38^ Moreover, the AQC-derivatization process introduced 10 C atoms into the amino compound molecule. We therefore needed to make a correction for naturally occurring C isotopes from the derivatization reagents to accurately estimate the atom % ^13^C of amino compounds in these derivatized molecules.^30,39^ This was done by calibrating the integrated atom % ^13^C from Orbitrap measurements against known ^13^C enrichment prepared by mixing of labeled and unlabeled amino acid mixtures. Isotopic calibration models were run for the whole ^13^C enrichment range (natural abundance to 98 atom % ^13^C) using linear regression, and for the low enrichment scale (natural abundance up to ∼2 to 5 atom % ^13^C) using curvilinear (quadratic, polynomial) regressions. Calibrations were performed on a compound-specific basis, given that they differed from compound to compound. For this reason, we next tried to find generalizations of isotope calibration models across compounds, based on the physicochemical properties of the single amino compounds. For this the polynomial terms a, b and c [polynom: f­(x) = a*x^2^ + b*x + c] of the single amino compounds were related to a range of molecular properties such as molecular weight, C:N ratio, C dilution through added AQC-C atoms, among others, and the resultant models used for predictions of isotope calibrations of the same compounds (model evaluation) and of unknowns (or uncalibrated knowns such as MurA). Then, the calibration model was evaluated by comparing the measured isotope calibration model against the predicted model using the regression coefficient (R^2^), mean absolute deviation (MAD), and mean absolute percentage deviation (MAPD) parameters. The detailed equations and descriptions are presented as “model evaluation” in the Supporting Information.

In a final step of sample isotope analysis, before calculating isotope enrichments of any amino compound, signal size dependent changes in isotope enrichment were corrected for. With decreasing signal intensity higher ^13^C isotopologues (e.g., *m*/*z* + 3, + 4) become unmeasurable which causes a systematic decline in isotope enrichments calculated from Orbitrap MS data at low signal intensities. Therefore, systematic offsets of atom % ^13^C in Orbitrap data were calculated relative to the 300 μM standard and corrected for in a concentration dependent manner for each amino acid (Figure S1). Only these data were then inserted into the isotope calibration models to obtain unbiased isotope enrichment numbers for each amino acid and sample. The estimations of the ^13^C isotopic LOD (LOD_isotope_) and LOQ (LOQ_isotope_) of the amino compounds were done by measuring the precision (standard deviation) of isotope enrichment (atom % ^13^C) in the natural abundance samples, as follows:

5 $${\mathrm{LOD}}_{\mathrm{isotope}}=3\times S_{n}$$

6 $${\mathrm{LOQ}}_{\mathrm{isotope}}=10\times S_{n}$$

where *S*_*n*_ refers to the standard deviation of atom % ^13^C in the natural abundance samples.^36^

Subsequent statistical analyses of the Achenkirch data set were performed using R version 4.2.2.^40^ First, the concentration and atom % ^13^C of all amino compounds were examined for outliers. Logarithmic or square root transformations were conducted if necessary to achieve homoscedasticity and normality. Significant differences in amino compounds between ^13^C-labeled and unlabeled soils were evaluated using Welch’s *t*-test. If homoscedasticity and normality were not obtained after transformation, a nonparametric Wilcoxon rank-sum test was performed.

## Results and Discussion

### Establishment of Standard Calibration Curves

The mass errors, retention times, linearities, LOD_concentration_, and LOQ_concentration_ for each amino compound were evaluated using concentration quantification standards ranging from 2.34 to 300 μM (Tables S1 and S2). Mass errors were calculated as the relative difference between the observed *m*/*z* values and the expected *m*/*z* values. The molecular ions of amino acid-AQC and amino sugar-AQC derivatives were detected at the expected *m*/*z* values, with the highest and average mass errors recorded at 2.79 and 0.67 ppm, respectively (Table S1). Notably, Lys and DAP can bind two AQC molecules due to their two primary amine groups, which we found at the expected *m*/*z* values (Table S1), but with peak areas similar to those of single-AQC derivatives. In contrast, Gln, Asn, Arg and His formed only single-AQC derivatives, despite containing more than one -NH group, as their side-chain N atoms belong to unreactive groups (amide-group in Gln and Asn, guanidino-group in Arg and imidazole-group in His). For simplicity in this study we therefore uniformly considered only the single-AQC derivatives. All amino acids exhibited distinct peaks at separate retention times within 15 min, successfully separating all isobaric amino acids, such as Ile and Leu. Additionally, all amino acids demonstrated excellent linearity between peak area and concentration (R^2^ > 0.991). This indicates that the separation of all standard amino acids was successfully achieved, along with the high precision and sensitivity of the AQC derivatization method using the UPLC-Orbitrap MS system. In terms of amino sugars, linearity was also excellent; hexosamines were well separated from MurA, but the three tested hexosamines, including GlcN, ManN, and GalN, overlapped and could not be separated in this method. AQC derivatization therefore quantifies the sum of hexosamines.

During sample analysis, we observed that the apparent atom % ^13^C of the respective amino compounds measured by Orbitrap at natural ^13^C abundance showed concentration dependency in the range of 2 to 300 μM amino compound, with declining atom % ^13^C at lower amino compound concentrations. This is caused by successive loss of detection of higher ^13^C isotopologues of amino acids with declining amino acid concentration, which systematically biases ^13^C content measurements in Orbitrap MS toward lower ^13^C enrichment values at smaller concentrations. Due to the varying contents of amino acids in the algal amino acid mixture and in environmental samples, we calculated the bias and corrected for this systematic error in isotope abundances. For natural ^13^C abundance standards we quantified the underestimation of atom % ^13^C at a specific concentration of the amino acid relative to a reference concentration. The reference concentration was set to 300 μM for each amino compound, i.e., the highest concentration used for concentration calibrations, where most quantitatively important higher isotopologues could be precisely quantified (mass error <5 ppm). The isotopic deviations ranged between 0.3 to 5 atom % ^13^C at lowest concentrations, depending on amino compound species. The deviation of measured atom % ^13^C to the reference was calibrated against concentration (using total peak area of this compound) and corrected for each amino compound in each standard and sample (Figure S1).

After this correction of concentration-dependent isotopic offset, we estimated the “true” atom % ^13^C of the respective amino compounds according to isotopic calibrations using mixed labeled and unlabeled algal amino acids. In the isotopic calibrations the measured Orbitrap-based atom % ^13^C values (*x*-axis) were related to their “true” ^13^C enrichment of the isotope standards (*y*-axis), and calibrations were divided into full isotopic ranges (1.1 to 99 atom % ^13^C, linear calibrations, Figure S2) and low isotope ranges (1.1 to ∼5 atom % ^13^C, Figure 1). Then, we established nonlinear fitting curves for all amino acid standards using a second-order polynomial equation (y = ax^2^ + bx + c) at low ^13^C enrichment levels, individually for each amino acid. Similar to the concentration dependency of the isotope bias, this nonlinearity was attributed to the incremental loss of detection of higher ^13^C isotopologues at lower ^13^C enrichment levels (though measured at the same concentration of the compound) during the Orbitrap mass spectrometric measurement. This phenomenon is caused by excessively low abundances of higher isotopologues (*m*/*z* values) of AQC-derivatives of amino compounds at decreasing ^13^C enrichment. Here, we only account for their monoisotopic (*m*/*z* 0) to higher isotopic forms (*m*/*z n*), where *n* represents the C numbers in respective amino compound molecule. For example, Gly has two C atoms, therefore the following isotopologue *m*/*z* values were used: 247.0949 (M0), 248.0982 (M1), 249.1016 (M2), while higher isotopologues originating from AQC addition were not observed in the low ^13^C enrichment range. The final isotope calibrations for each amino acid exhibited excellent regression fitting (polynomial R^2^ > 0.990), but with distinct polynomial fitting terms for each amino acid, including the quadratic terms “a”, the linear terms “b”, and the constant terms “c” (Figure 1). These isotope curves enabled us to calibrate the atom % ^13^C of various amino acid-AQC derivatives measured by Orbitrap to the ‘true’ atom % ^13^C of the corresponding amino acids.

**Figure 1:**
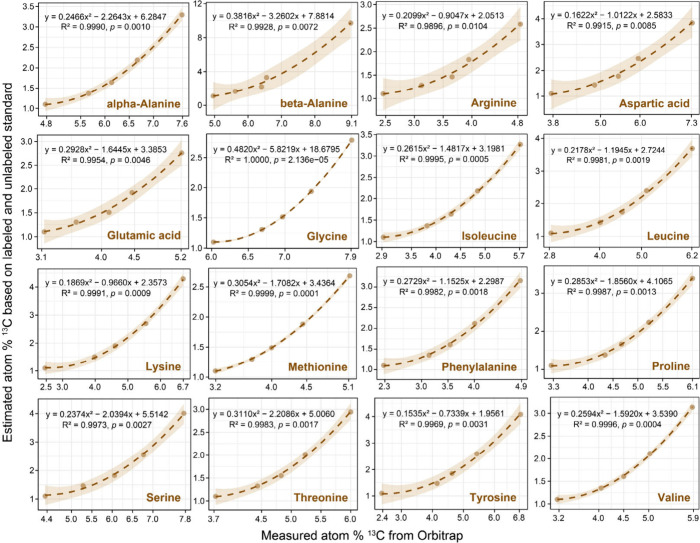
Isotope calibration curves of individual standard amino acids based on second-order polynomial functions (y = ax^2^ + bx + c).

### Development of Predictive Model

Based on the polynomial fitting terms of the different amino acid isotope standards, we developed equations to relate the calibrated regression terms to chemical properties of the respective amino acids and their AQC-derivatives. The fold dilution of carbon numbers (FD_C_) was calculated as total C atoms (C_tot_) summed from the amino acid (C_AA_) and the C atoms deriving from the AQC reagent (AQC adds 10 C atoms per amino acid; C_tot_ = C_AA_ + 10), divided by the C atom number of the native amino acid (C_AA_). FDc values varied among different amino acids and ranged from 6 in glycine to 2.11 in tyrosine or phenylalanine. We first performed a linear regression of the Orbitrap-derived ^13^C atom % for unlabeled standards against FD_C_, demonstrating great linearity (R^2^ = 0.973) between the two (Figure 2a). No other tested molecular property (molecular weight or C:N ratio of amino compounds) better predicted Orbitrap measured atom % ^13^C of amino acids at natural isotope abundance which is defining the c (intercept) value of the polynomial isotope regressions and therefore is the prime parameter of isotope calibrations at very low enrichment levels, indicating zero isotope enrichment and therefore unlabeled controls. This regression enabled us to accurately predict Orbitrap natural ^13^C abundance signals for other amino compounds, such as GlcN, MurA, Hyp, GAB, and DAP, which were either unavailable or are prohibitively expensive as isotopically labeled standards.^6,14^

**Figure 2:**
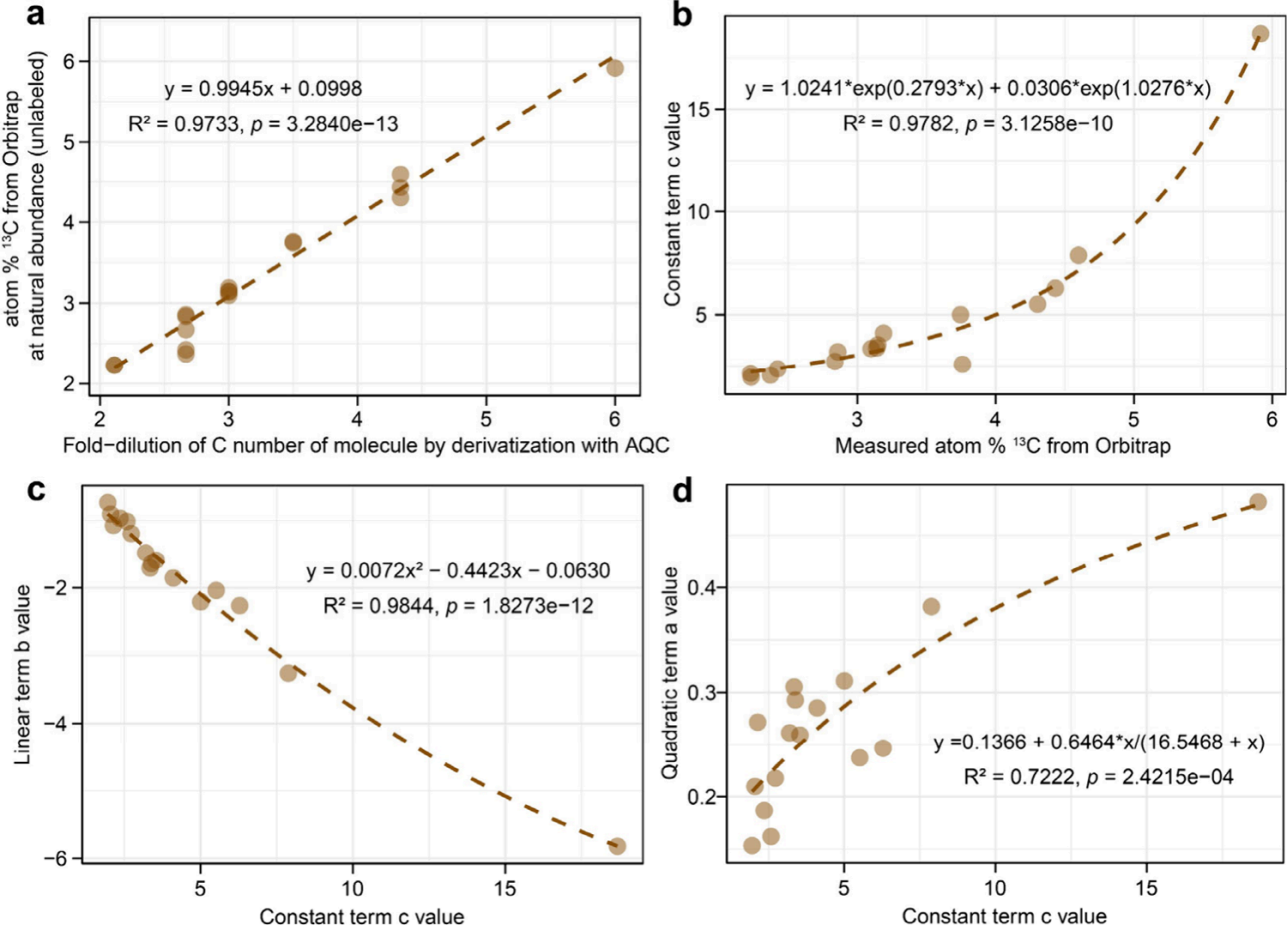
Regression curves between (a) fold-dilution of C number of derivatized molecules and atom % ^13^C from Orbitrap at natural abundance, (b) atom % ^13^C from Orbitrap at natural abundance and constant terms c values, (c) constant terms c values and linear terms b values, and (d) constant terms c values and quadratic terms a values, based on measurements of unlabeled amino acid standards.

Then, we performed a nonlinear regression between the constant terms c values of the isotopic standard curves and the Orbitrap-derived ^13^C atom % of unlabeled standards, to best constrain the baseline of the natural isotope abundance, i.e. with “zero” or no isotope enrichment which also showed excellent regression performance (R^2^ = 0.978) (Figure 2b). Likewise, we used the predicted natural ^13^C abundance for those amino compounds that were unavailable as isotopically labeled standards to predict their constant c values based on the second regression equation. Subsequently, we performed two further nonlinear regressions to predict the other polynomial regression terms: one relating the linear terms b to the constant terms c (R^2^ = 0.984), and another one relating the quadratic terms a to the constant terms c (R^2^ = 0.722) for the known amino acid standards (Figure 2c and d). Using these regressions, we predicted the quadratic terms a and linear terms b for the amino compounds lacking isotopically labeled standards, thereby developing the general predicted isotope calibration curves for these compounds (Figure 3). Though we tested for other predictors of the polynomial regression terms b and c we did not find better approaches, and therefore accepted the consequential uncertainty inflation caused by using predicted values of c to finally predict b and a values. In this final correlation process, Gly showed the highest values for the Orbitrap-derived ^13^C atom %, as well as for the quadratic term a, the linear term b, and the constant term c among the amino acid standards. This is attributed to Gly’s highest FD_C_ value, which is due to its minimal carbon atom count being the smallest amino acid. In addition, the predicted atom % ^13^C for each isotope calibration curve approached 1.1 atom % ^13^C natural abundance after isotope calibration.

**Figure 3:**
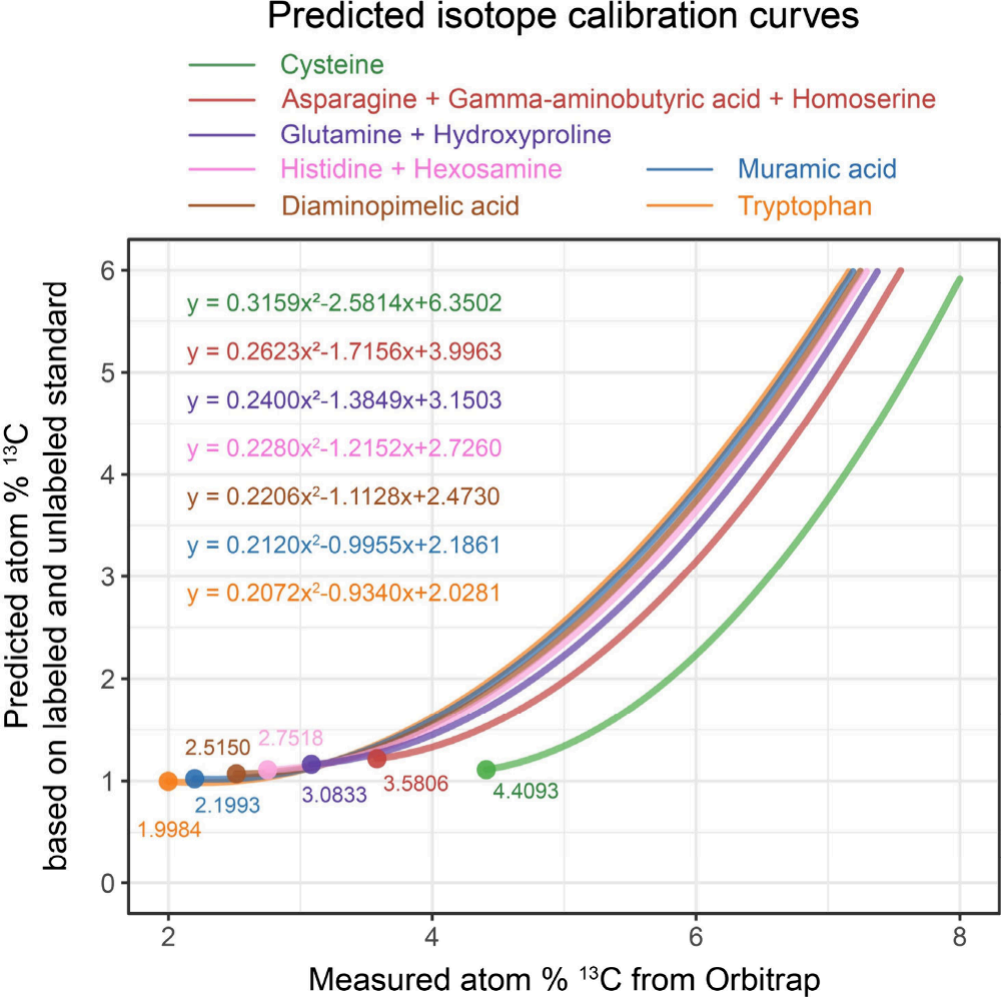
Predicted isotope calibration curves for those amino compounds unavailable as ^13^C labeled standards under conventional acid hydrolysis condition, based on equations developed in Figure 1, based on different values of fold-dilution of C number of the AQC-derivatized molecules. Starting dots indicate the natural ^13^C abundance of amino compound-AQC derivatives from Orbitrap. Black dashed line indicates the natural ^13^C abundance (1.1 atom % ^13^C).

In Figure 3, we display predicted calibration curves for Cys, Asn, GAB, Hse, Gln, Hyp, His, hexosamines (including GlcN, GalN, and ManN), DAP, MurA, and Trp derived from our model. Each curve had a distinct polynomial equation due to the unique carbon dilutions of the different amino compounds. This same model is applicable to other amino compounds as well not studied here. Currently, with over 500 naturally occurring amino acids (and 20+ amino sugars), analyzing the wide range of proteinogenic and nonproteinogenic amino acids remains an enormous challenge due to their diverse physicochemical properties and the lack of analytical standards,^41^ not to mention the added complexity of isotope enrichment analysis. We need to mention that although we developed the method based on ^13^C isotope standards in this study, it is also applicable to ^15^N isotopologues; however, the calibrations need to be rerun, and the correlations between physicochemical properties of amino compounds and the polynomial calibration terms need to be reassessed. Moreover, we are certain that the predictive model can be further improved based on implementing a wider range of physicochemical properties (isoelectric point, molecular polarity, or functional group composition) of the AQC-derivatives, molecular features that we likely did not consider thus far, but may affect the derivatization efficiency and ionization behavior for mass spectrometric analysis.

### Model Validation

To assess the accuracy of the general isotope calibration model, we inserted the FD_C_ values of those amino acids for which we had ^13^C labeled standards and predicted their calibration curves using the series of regression equations described above. We therefore obtained two calibration curves (isotope standard-based and therefore measured, and model-based predicted) for each known, calibrated amino acid, corresponding to the five lowest ^13^C enrichment standards that we measured. As illustrated in the conceptual sketch of model comparison (Figure S3a), we compared the difference between the original *y*-axis values of the standard curves and the predicted *y*-axis values of the predicted curves. Linear regression, a common and straightforward method for evaluating model accuracy, was used to compare observed and predicted values. The slope and intercept of this regression was analyzed against the 1:1 line to determine whether the intercept was close to 0 and the slope close to 1 (i.e., the ideal regression as y = x; Figure S3b).^42,43^ Across all available compounds we obtained a slope of 1.019 (indicating less than 2% relative deviation from the 1:1 line), an intercept close to 0, i.e. 0.168, and R^2^ of 0.884. Additionally, we assessed the predictive performance of our model through R^2^, MAD, and MAPD for each single amino compound (Table S3). Higher R^2^ and lower MAD and MAPD values indicate better prediction performance.^44^ The parameter MAPD, expressed as a percentage (%), is a statistical parameter for model accuracy that is particularly useful because it does not depend on the actual magnitude of the dependent variable.^45^ The linear regressions of the atom % ^13^C values of single amino acids from original calibration and predicted calibration resulted in R^2^ of >0.97, with respective average MAD and MAPD values being 0.334 and 15.8% (Table S3). These results demonstrated the high effectiveness of predicting atom % ^13^C of amino acid-AQC derivatives for individual amino acids at natural abundance and in the low ^13^C enrichment range. Across the whole isotope range (1.1 to 99 atom % ^13^C) calibration models became linear (Figure S2) and therefore are likely easier to predict (but this was not the target in this study).

Additionally, we conducted linear regressions of atom % ^13^C values from original calibration against the predicted calibration for each individual amino acid. Notably, the MAPD values for all amino acids were below 50%, falling into three categories: excellent (MAPD < 10%), good (10% ≤ MAPD ≤ 20%), and reasonable (MAPD ≤ 50%).^45,46^ Specifically, Glu (5.7%), Ile (2.4%), Leu (5.87%), Lys (5.8%), Met (6.4%), and Val (7.7%) were in the excellent category; β-Ala (, 14.4%), Arg (11.0%), Phe (17.9%), Pro (12.0%), Ser (19.1%), Thr (12.8%), and Tyr (17.0%) in the good category; and α-Ala (37.9%), Asp (29.5%), and Gly (47.2%) in the reasonable category. For amino acids not classified as excellent, if the regression line was above the ideal regression (y = x), the prediction was considered an overestimate; if below, it was considered an underestimate. MAD indicated the average magnitude of overestimation or underestimation of the original atom % ^13^C values. We observed that the predicted atom % ^13^C for α-Ala, Asp, Gly, Pro, Ser, Thr, and Tyr were overestimated relative to the original values, while β-Ala, Arg, and Phe were underestimated (Table S3). This indicates that the model performs well at low isotope enrichment levels and is particularly effective for use in biogeochemical studies. While over- and underestimates in atom % ^13^C can occur for amino acids that are not directly calibrated isotopically but need to be predicted, in isotope tracing studies the ^13^C enrichment is measured against the natural ^13^C abundance (control, no isotope amendment). Here, these overestimates or underestimates are calculated out, as atom % excess is applied for further calculations, and this is derived as the difference in atom % ^13^C of the ^13^C labeled sample minus the atom % ^13^C of the unlabeled control. This effectively cancels out any bias as long as linearity is given and the slope is close to 1 between predicted and original isotope calibrations.

Many current studies of soil microbial C cycling have targeted realistic C input amounts, which results in low isotope enrichment levels under near-native soil conditions. On the other hand, many previous studies had only access to C substrates (e.g., plant necromass, root exudates) as input with low ^13^C enrichment (∼4 atom % ^13^C),^47–49^ leading to very low ^13^C incorporation levels into soil amino compounds. In both cases of low isotope incorporation, isotope biogeochemists face the trade-off between the excellent isotope precision of compound-specific IRMS and the high C amounts needed per injection, accompanied by low chromatographic resolution when coupled to IRMS, aside of eventual needs to derivatize compounds for gas chromatographic separation.^50,51^ Only separate protocols exist to determine C isotopes in amino acids and in amino sugars by GC-IRMS and/or LC-IRMS.^50,51^ In contrast, the LC-Orbitrap MS combines the unique separation efficiency of UPLC with the very low detection limits and low C needs of Orbitrap MS. Yet, the isotope precision has been formerly discussed to be relatively low for Orbitrap systems (0.1–1.0 atom %).^52–54^ Only recently we showed high precision for UPLC-Orbitrap MS in the isotope labeling range (∼0.03 atom %),^18^ and others even demonstrated precision for natural abundance isotope measurements in oxoanions if *m*/*z* signals are integrated for minutes to hours (0.001 atom %).^55–57^ Therefore, with the isotope calibrations as optimized here a gap can be filled allowing precise isotope measurements in LC peaks eluting in a few seconds up to fraction of a minute, with great amount reductions relative to IRMS. In addition, our general isotope calibration model performs well and can be effectively used to predict the atom % ^13^C in a much wider range of amino compounds in future research. Because this method is based on a statistical model of molecular structure (i.e., the proportion of C atoms in AQC derivatives deriving from the original compound) explaining isotope response curves, it should be transferable to other high-resolution MS platforms such as LC-TOF/MS and GC-TOF/MS, provided that the target derivatives and isotopologues are well resolved and new ^13^C-labeled and unlabeled calibration sets are established to account for platform-specific differences. However, TOF systems operate lower than Orbitrap mass resolution and therefore may be limited by background noise, sample complexity, and isotope sensitivity. Standard LC-TOF/MS systems usually reach an isotope precision of around 0.1–1 atom %, while high-end HR-TOF instruments (with mass resolutions of 30,000+) can lower this to about 0.05–0.1 atom % under ideal conditions, depending on *m*/*z* and sample complexity.^58,59^

### ^13^C Enrichment Analysis in Soil

With real forest soil samples from an *in situ* ^13^C labeling experiment, we assessed (i) the precision of isotope measurements by Orbitrap MS in low ^13^C enrichment and natural isotope abundance range, and (ii) the applicability of the measured versus predicted calibration models for (potentially) important biomarker amino compounds. All target amino compounds were clearly quantifiable (mass error <5 ppm, LOD_concentration_ ranging from 0.12 to 8.64 μmol L^–1^, linearity R^2^ > 0.991), and we therefore compared the concentration and atom % ^13^C enrichment of different amino compounds between unlabeled control soils (water addition) and soils amended with realistically low amounts of ^13^C labeled root exudate mimics. We first performed the concentration-dependent correction of isotope offsets at low amino compound contents before we applied the ^13^C isotope calibration models (measured or predicted).

Regarding the concentration results, Glu was the most abundant amino acid observed in all soil samples, whereas Met was the least abundant (Figure 4a). Among amino sugars, hexosamines (GlcN, GalN, and ManN; were not separated here and therefore are treated as a sum) were significantly more abundant than MurA. Importantly, GlcN and MurA are crucial structural components of microbial cell walls and are commonly used as biomarkers for microbial necromass in soils.^60^ Our results showed that the AQC derivatization method is practicable for the simultaneous analysis of amino acids and amino sugars. Meanwhile, we found no significant differences in the concentration of bound amino compounds between unlabeled and labeled soil samples except for mDAP (Figure 4a), indicating that the addition of low exudate amounts did not alter microbial assimilation and accumulation of amino compounds in bulk soils.

**Figure 4:**
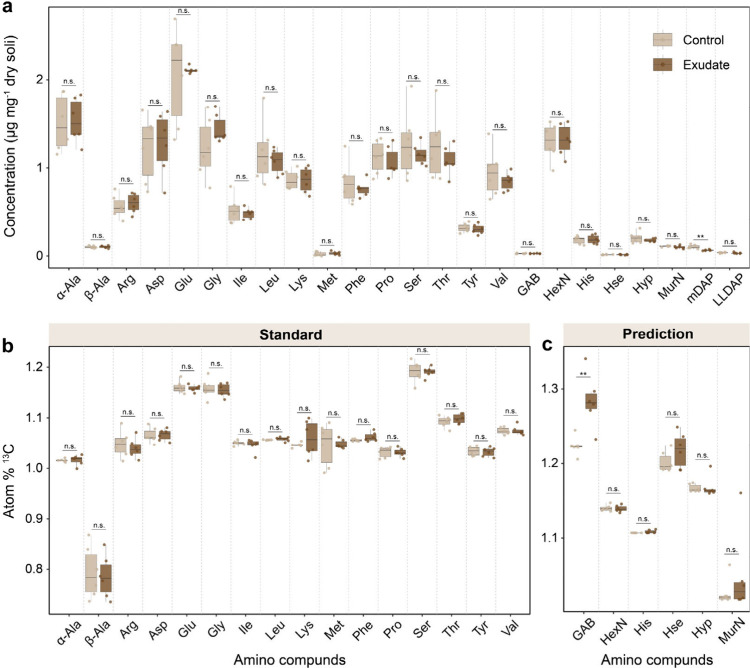
(a) The contents of all amino compounds, paired for controls (light colored boxes) and ^13^C amended soils (dark colored boxes) (*n* = 6). The atom % ^13^C of amino compounds deriving from (b) standard calibrations and (c) predicted calibrations, paired for controls and ^13^C amended soils. The significance levels are indicated with asterisks and n.s. (*p < 0.05; **p < 0.01; ***p < 0.001; n.s., not significant).

Furthermore, the atom % ^13^C of all amino compounds was calculated using both the standard isotope calibration curves (Figure 4b) and the predicted isotope calibration curves (Figure 4c). We observed that the isotope values for both measured and predicted calibrations fluctuated around 1.1 atom % ^13^C, which well reflects the average natural ^13^C abundance of soils. To assess the overall LC-Orbitrap MS precision for soils we calculated the standard deviations (SD) of atom % ^13^C of all amino compounds in unlabeled control samples (Table S4). These ranged from 0.0001 to 0.05 atom % ^13^C, with an average of 0.012%. Using 3-times and 10-times the SD measured at natural isotope abundance provides the LOD_isotope_ (ranging from 0.0003 to 0.14 atom % ^13^C) and LOQ_isotope_ (ranging from 0.0010 to 0.47 atom % ^13^C) for isotope incorporation measurement (Table S4). These LODs are excellent given the short temporal integration window available in UPLC-MS measurements, though certainly not as low as those known from LC-IRMS and GC-IRMS. Substantial matrix effects are expected to cause decreases in the precision of isotope measurements and therefore increases in LOD_isotope_.^61^ Hence, we also compared the LOD_isotope_ values from standards (no matrix effect, Table S2) to those from soils (including potential soil matrix effects, Table S4). The relationship between LOD_isotope_ of standards and soils was shown in Figure S4, where soil values plot at or below the 1:1 line for most amino compounds, indicating no negative effect on isotope precision in samples compared to standards. Actually we found lower LOD_isotope_ values in soils than in standards. For mDAP and LLDAP, we could not detect the abundance of their higher isotopologues but only detected their monoisotopic forms (*m*/*z* 0) due to low mass signal intensities, so that their atom % ^13^C values could not be calculated. There were minimal differences in atom % ^13^C for most amino compounds between controls and ^13^C amended soils, with significant ^13^C enrichment observed only for GAB. The predicted calibration model therefore worked effectively to calibrate atom % ^13^C for unknown or uncalibrated amino compounds such as GAB. It is worth noting that the framework we propose here is a new approach to trace isotopes across environmentally important amino compounds, including primary and secondary amines, potentially in any kind of matrix—not limited to soils. It can be applied to other complex matrices such as sediments and water columns,^62,63^ plant tissues and microbial isolates,^64^ and clinical or technical samples,^65^ particularly where high-resolution isotopologue separation and low isotope incorporation levels are of interest to trace the formation and sink processes of amino compounds. We are fully aware that the calibration models might be further improved by isotopically measuring and calibrating more amino compounds, and by using even better constraints of the polynomial regression terms with added physicochemical traits of these compounds. However, this was outside of the scope of this study here. Isotopically calibrating the compounds of interest directly using mixtures of labeled and unlabeled pure standards remains the “gold standard”, though the proposed prediction of isotope calibration models can help fill gaps until a wider availability of important isotope standards becomes reality.

## Conclusions

The study developed a comprehensive and ultrasensitive isotope calibration method (LOD_concentration_ ranging from 0.12 to 8.64 μM and LOD_isotope_ ranging from 0.0003 to 0.14 atom %) for the soil amino compound profile using UPLC Orbitrap-MS. This method effectively corrects for isotope abundance interference from derivatization reagents, for signal size dependent underestimations of ^13^C abundance, and enables the simultaneous separation and quantification of amino acids and amino sugars through AQC derivatization. Additionally, it allows us to predict the ^13^C calibrations of amino compounds that are unavailable as isotopically labeled standards, providing accurate isotope calibration curves for quantifying isotope enrichments across the entire soil amino compound profile. Model validation using R^2^, MAD, and MAPD parameters demonstrated the good accuracy of the predictive model. Examination of soil samples confirmed the applicability and high isotope sensitivity of our method. Overall, our proposed approach offers valuable insights for a better understanding of the dynamics and fate of different amino compounds in soils and other complex environmental systems.

## Supplementary Material



## Data Availability

The data set and all R codes used to produce the analysis and figures in this study can be found on Zenodo (https://doi.org/10.5281/zenodo.15564201). The Orbitrap MS RAW data files, including those of all samples, the isotope calibration standards of U-^13^C labeled and unlabeled algal amino acid mixtures, and soil samples, were deposited in MetaboLights (EMBL-EBI, accession number MTBLS12550).

## Notes

Calibration model evaluation descriptions; chemical information on measured compounds (Table S1); MS validation performance metrics on standards (Table S2); comparisons of model evaluation parameters (Table S3); standard deviations (SD) of atom % ^13^C of all compounds (Table S4); concentration dependent isotopic offsets of different amino acids (Figure S1); linear isotope calibration curves of standard amino acids (Figure S2); results of linear regression in model evaluation (Figure S3); matrix effect evaluation between standard and soil samples (Figure S4) (PDF)
