# Supplementary material for: A Comprehensive and Ultrasensitive Isotope Calibration Method for Soil Amino Compounds Using Orbitrap Mass Spectrometry
Source: Anal Chem. 2025 Jun 12;97(24):12679–89. doi: 10.1021/acs.analchem.5c01358 (PMC12199224; doi:10.1021/acs.analchem.5c01358)
Supplement: Supplementary file 1 [file ac5c01358_si_001.pdf]

Supporting Information for

A Comprehensive and Ultrasensitive Isotope Calibration Method for Soil Amino Compounds Using Orbitrap Mass Spectrometry

Tao Li[a,b,*], Yuhua Li[a,b], Erika Salas[a,b], Ye Tian[a,b,c], Xiaofei Liu[a,b,d], and Wolfgang Wanek[a,*]

[a] Division of Terrestrial Ecosystem Research, Department of Microbiology and Ecosystem Science, Centre for Microbiology and Environmental Systems Science, University of Vienna, Djerassiplatz 1, A-1030 Vienna, Austria

[b] Doctoral School in Microbiology and Environmental Science, University of Vienna, Djerassiplatz 1, A-1030 Vienna, Austria

[c] Department of Soil and Environment, Swedish University of Agricultural Sciences, Lennart Hjelms väg 9, 756 51 Uppsala, Sweden

[d] State Key Laboratory for Subtropical Mountain Ecology of the Ministry of Science and Technology and Fujian Province, Fujian Normal University, Fuzhou 350007, China

*Corresponding author

Tao Li:

tao197@univie.ac.at

Wolfgang Wanek:

wolfgang.wanek@univie.ac.at

TABLE OF CONTENTS

**Model Evaluation** Calibration model evaluation descriptions	**S3**
**Table S1** Chemical information of measured compounds	**S4-5**
**Table S2** MS validation performance metrics on standards	**S6**
**Table S3** Comparisons of model evaluation parameters	**S7**
**Table S4** Standard deviations (SD) of atom % ^{13}C of all compounds	**S8**
**Figure S1** Concentration dependent isotopic offsets of different amino acids	**S9**
**Figure S2** Linear isotope calibration curves of standard amino acids	**S10**
**Figure S3** Results of linear regression in model evaluation	**S11**
**Figure S4** Matrix effect evaluation between standard and soil samples	**S12**
**References**	**S13**

Model Evaluation

The calibration model was evaluated by comparing the measured isotope calibration model against the predicted model using R^2, MAD, and MAPD as follows:

$$R^2 = 1 - \frac{\sum_{i=1}^{m} (Y_i - X_i)^2}{\sum_{i=1}^{m} \left(Y_i - \overline{Y}_i\right)^2} \tag{1}$$

$$MAD = \frac{1}{m} \sum_{i=1}^{m} |Y_i - X_i| \tag{2}$$

$$MAPD = \frac{1}{m} \sum_{i=1}^{m} \left| \frac{Y_i - X_i}{Y_i} \right| \times 100\% \tag{3}$$

where m is the total number of datasets, X_i is the ith predicted value from the standard calibration curves, Y_i is the ith original value from the standard calibration model, and $\overline{Y}_i$ is the average of the original values from the standard calibration model. R^2 indicates the goodness of fit of the model, ranging from 0 to 1. MAD stands for mean absolute deviation and is also commonly referred to as mean absolute error (MAE). It represents the average magnitude of the prediction deviations. MAPD, often referred to as mean absolute percentage error (MAPE), is also a widely used parameter for model evaluation.[1] It can be categorized into four levels: excellent (MAPD < 10 %), good (10% ≤ MAPD ≤ 20%), reasonable (20% ≤ MAPD ≤ 50%), and inaccurate (MAPD > 50%).[2,3] High model accuracy is indicated by low values of MAD and MAPD, as well as a high R^2 value.[4]

Table S1. Abbreviations, chemical formulae, fold-dilution of carbon number (FD_C), retention times, detected masses, mass accuracies, linearities, and isotopic limits of detection ($LOD_{isotope}$) of all unlabeled standard amino compounds measured by UPLC-Orbitrap MS.

Compound	Abbreviation	Chemical formula	FD_C	Retention time (min)	*m*/*z* $[M+H]^+$	Expected AQC derivatives $[M+H]^+$	Observed AQC derivatives $[M+H]^+$	Mass error (ppm)
alpha-Alanine	α-Ala	$C_3H_7NO_2$	4.33	6.49	90.05550	261.11051	261.11082	+1.18
beta-Alanine	β-Ala	$C_3H_7NO_2$	4.33	5.95	90.05550	261.11051	261.11049	−0.08
Arginine	Arg	$C_6H_{14}N_4O_2$	2.67	2.99	175.11950	346.17451	346.17451	−0.01
Aspartic acid	Asp	$C_4H_7NO_4$	3.50	5.45	134.04533	305.10034	305.10066	+1.05
meso-2,6-diaminopimelic acid*	mDAP	$C_7H_{14}N_2O_4$	2.43	6.92/7.07	191.10318	362.15819	362.15831	+0.33
LL-2,6-diaminopimelic acid*	LLDAP	$C_7H_{14}N_2O_4$	2.43	7.07	191.10318	362.15819	362.15791	−0.77
gamma-Aminobutyric acid	γ-GAB	$C_4H_{10}N_2O$	3.50	6.57	104.07116	275.12617	275.12599	−0.65
Glutamic acid	Glu	$C_5H_9NO_4$	3.00	5.84	148.06099	319.11600	319.11656	+1.75
Glycine	Gly	$C_2H_5NO_2$	6.00	4.87	76.03985	247.09486	247.09555	+2.79
Hexosamine	HexN	$C_6H_{13}NO_5$	2.67	0.96	180.08720	351.14221	351.14177	−1.25
Histidine	His	$C_6H_9N_3O_2$	2.67	1.06	156.07730	327.13231	327.13231	−0.01
Homoserine	Hse	$C_4H_9NO_3$	3.50	5.10	120.06607	291.12108	291.12169	+2.10
Hydroxyproline	Hyp	$C_5H_9NO_3$	3.00	1.85	132.06607	303.12108	303.12111	+0.10
Isoleucine	Ile	$C_6H_{13}NO_2$	2.67	9.98	132.10246	303.15747	303.15794	+1.55
Leucine	Leu	$C_6H_{13}NO_2$	2.67	10.14	132.10246	303.15747	303.15751	+0.13
Lysine*	Lys	$C_6H_{14}N_2O_2$	2.67	7.77	147.11336	318.16837	318.16829	−0.25
Methionine	Met	$C_5H_{11}NO_2S$	3.00	8.45	150.05888	321.11389	321.11418	+0.90
Phenylalanine	Phe	$C_9H_{11}NO_2$	2.11	10.32	166.08680	337.14181	337.14210	+0.86

Proline	Pro	$C_5H_9NO_2$	3.00	6.98	116.07116	287.12617	287.12657	+1.39
Serine	Ser	$C_3H_7NO_3$	4.33	4.15	106.05042	277.10543	277.10557	+0.51
Threonine	Thr	$C_4H_9NO_3$	3.50	6.17	120.06607	291.12108	291.12129	+0.72
Tyrosine	Tyr	$C_9H_{11}NO_3$	2.11	8.18	182.08172	353.13673	353.13707	+0.96
Valine	Val	$C_5H_{11}NO_2$	3.00	8.67	118.08680	289.14181	289.14216	+1.21
Muramic acid	MurA	$C_9H_{17}NO_7$	2.11	5.36/6.16	252.10832	423.16333	423.16398	+1.53

Notes: 1. HexN includes glucosamine (GlcN), galactosamine (GalN), and mannosamine (ManN). 2. * Lys (488.21555) and mDAP/ LLDAP (532.20537) derivatives with two AQC were observed at the expected *m*/*z* $[M+H]^+$ values. No multi-AQC derivatives were, however, observed for Asn, Gln, His and Arg though these amino acids also contain multiple -NH groups.

Table S2. Linearities, dynamic range, limits of detection in concentration ($LOD_{concentation}$), limits of quantification in concentration ($LOQ_{concentration}$), isotopic limits of detection ($LOD_{isotope}$), and isotopic limits of quantification ($LOQ_{isotope}$) of all standard amino compounds measured by UPLC-Orbitrap MS.

**Compound**	**Linearity (R^2)**	**Dynamic range (μM)**	**$LOD_{concentration}$ (μM)**	**$LOQ_{concentration}$ (μM)**	**$LOD_{isotope}$ (atom %)**	**$LOQ_{isotope}$ (atom %)**
alpha-Alanine	0.9997	2.34–300	0.35	1.16	0.16	0.53
beta-Alanine	0.9996	2.34–300	0.19	0.63	0.47	1.57
Arginine	0.9909	2.34–300	0.81	2.69	0.07	0.23
Aspartic acid	0.9975	2.34–300	0.63	2.08	0.05	0.17
meso-2,6-diaminopimelic acid	0.9993	9.38–300	6.94	23.15	−	−
LL-2,6-diaminopimelic acid	0.9974	9.38–300	8.64	28.80	−	−
gamma-Aminobutyric acid	0.9987	2.34–300	0.34	1.12	−	−
Glutamic acid	0.9999	2.34–300	0.48	1.59	0.04	0.13
Glycine	0.9995	2.34–300	0.98	3.25	0.09	0.30
Hexosamine	0.9987	2.34–300	0.34	0.79	−	−
Histidine	0.9918	2.34–300	2.04	6.80	0.04	0.13
Homoserine	0.9997	2.34–300	0.45	1.50	−	−
Hydroxyproline	0.9999	2.34–300	0.59	1.97	−	−
Isoleucine	0.9999	2.34–300	0.24	0.79	0.27	0.90
Leucine	0.9977	2.34–300	0.28	0.94	0.02	0.07
Lysine*	0.9997	2.34–300	2.61	8.70	0.12	0.40
Methionine	0.9993	2.34–300	0.33	1.10	0.12	0.40
Phenylalanine	0.9994	2.34–300	0.12	0.40	0.04	0.13
Proline	0.9991	2.34–300	0.32	1.07	0.15	0.50
Serine	0.9991	2.34–300	0.72	2.39	0.34	1.13
Threonine	0.9997	2.34–300	0.33	1.10	0.19	0.63
Tyrosine	0.9999	2.34–300	0.44	1.47	0.10	0.33
Valine	0.9994	2.34–300	0.22	0.75	0.06	0.20
Muramic acid	0.9992	2.34–300	2.03	6.76	−	−

Notes: − lack of isotopic labeled standards or undetected here, e.g., the intensity of heavy isotopic peak area in DAP was too low to calculate compared with other amino compounds at natural abundance.

Table S3. Comparisons of regression coefficient (R^2), mean absolute deviation (MAD), and mean absolute percent deviation (MAPD%) between original isotope calibration models and predicted isotope calibration models. The original models were isotope standard calibration curves of the respective amino acids, and test models were established by input fold-dilution of C number (FD_C) from the corresponding amino acid into our general predicted isotope calibration curves.

Compounds	Performance	Accuracy evaluation		
		R^2	MAD	MAPE (%)
alpha-Alanine	Overestimate	0.9946	0.8068	37.90
beta-Alanine	Underestimate	0.9926	0.4041	14.43
Arginine	Underestimate	0.9744	0.2040	10.97
Aspartic acid	Overestimate	0.9912	0.6983	29.45
Glutamic acid	**Excellent**	0.9952	**0.1157**	**5.70**
Glycine	Overestimate	0.9956	0.7100	47.18
Isoleucine	**Excellent**	0.9986	**0.0404**	**2.42**
Leucine	**Excellent**	0.9980	**0.1313**	**5.87**
Lysine	**Excellent**	0.9990	**0.1839**	**5.79**
Methionine	**Excellent**	0.9996	**0.1253**	**6.38**
Phenylalanine	Underestimate	0.9955	0.3741	17.90
Proline	Overestimate	0.9945	0.2168	12.03
Serine	Overestimate	0.9964	0.5359	19.11
Threonine	Overestimate	0.9960	0.2072	12.78
Tyrosine	Overestimate	0.9969	0.4484	16.99
Valine	**Excellent**	0.9985	**0.1349**	**7.70**

Table S4. Standard deviations (SD) of atom % ^{13}C of all compounds, and their isotopic limits of detection ($LOD_{isotope}$) and isotopic limits of quantification ($LOQ_{isotope}$) at natural isotopic abundance in unlabeled soil samples.

**Compounds**	**SD of atom % ^{13}C**	**$LOD_{isotope}$ (atom %)**	**$LOQ_{isotope}$ (atom %)**
alpha-Alanine	0.0028	0.0084	0.0280
beta-Alanine	0.0472	0.1415	0.4720
Arginine	0.0242	0.0726	0.2420
Aspartic acid	0.0136	0.0408	0.1360
gamma-Aminobutyric acid	0.0114	0.0343	0.1140
Glutamic acid	0.0110	0.0331	0.1100
Glycine	0.0174	0.0522	0.1740
Hexosamine	0.0038	0.0114	0.0380
Histidine	0.0001	0.0003	0.0010
Homoserine	0.0126	0.0379	0.1260
Hydroxyproline	0.0047	0.0141	0.0470
Isoleucine	0.0043	0.0129	0.0430
Leucine	0.0004	0.0013	0.0040
Lysine	0.0043	0.0128	0.0430
Methionine	0.0386	0.1157	0.3860
Phenylalanine	0.0021	0.0064	0.0210
Proline	0.0096	0.0288	0.0960
Serine	0.0190	0.0571	0.1900
Threonine	0.0100	0.0300	0.1000
Tyrosine	0.0083	0.0248	0.0830
Valine	0.0062	0.0187	0.0620
Muramic acid	0.0165	0.0494	0.1650

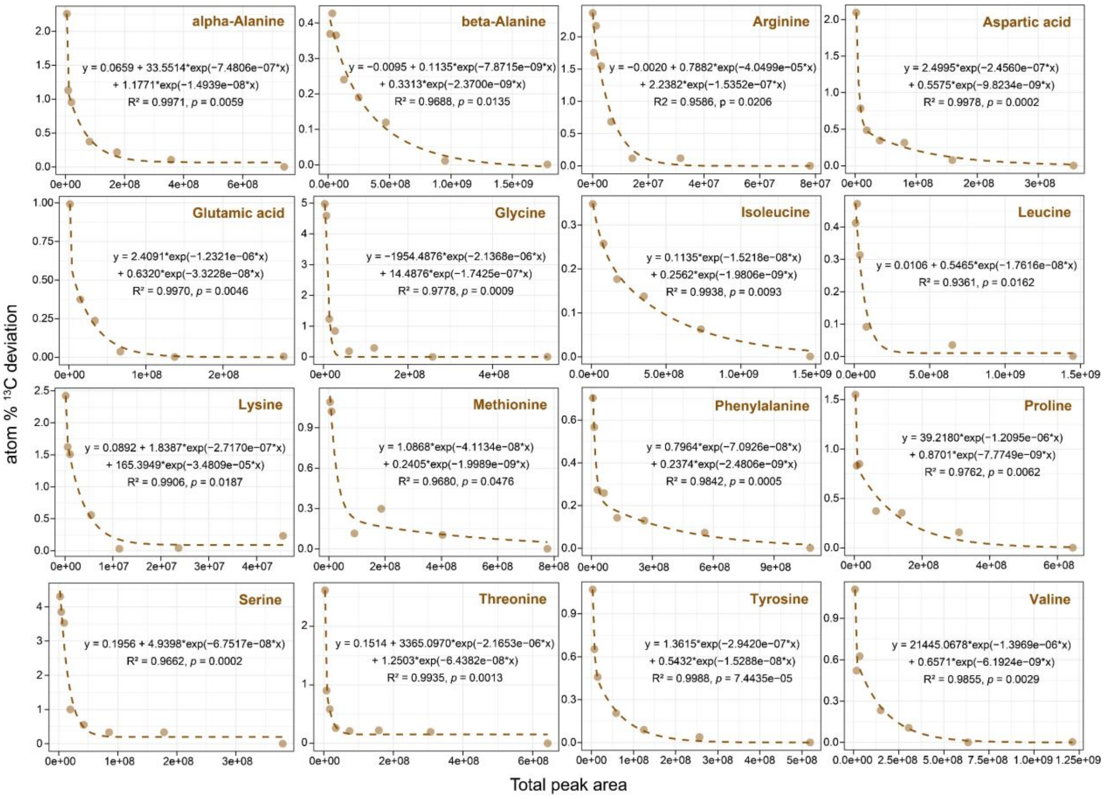

Figure S1. Concentration dependent isotopic offsets of different standard amino acids. Curve fitting was carried out based on the deviation between the atom % ^{13}C at 300 μM concentration of each amino acid standard and the atom % ^{13}C at concentrations lower than the target (300 μM). Concentrations here are represented by the total peak area measured for each corresponding standard.

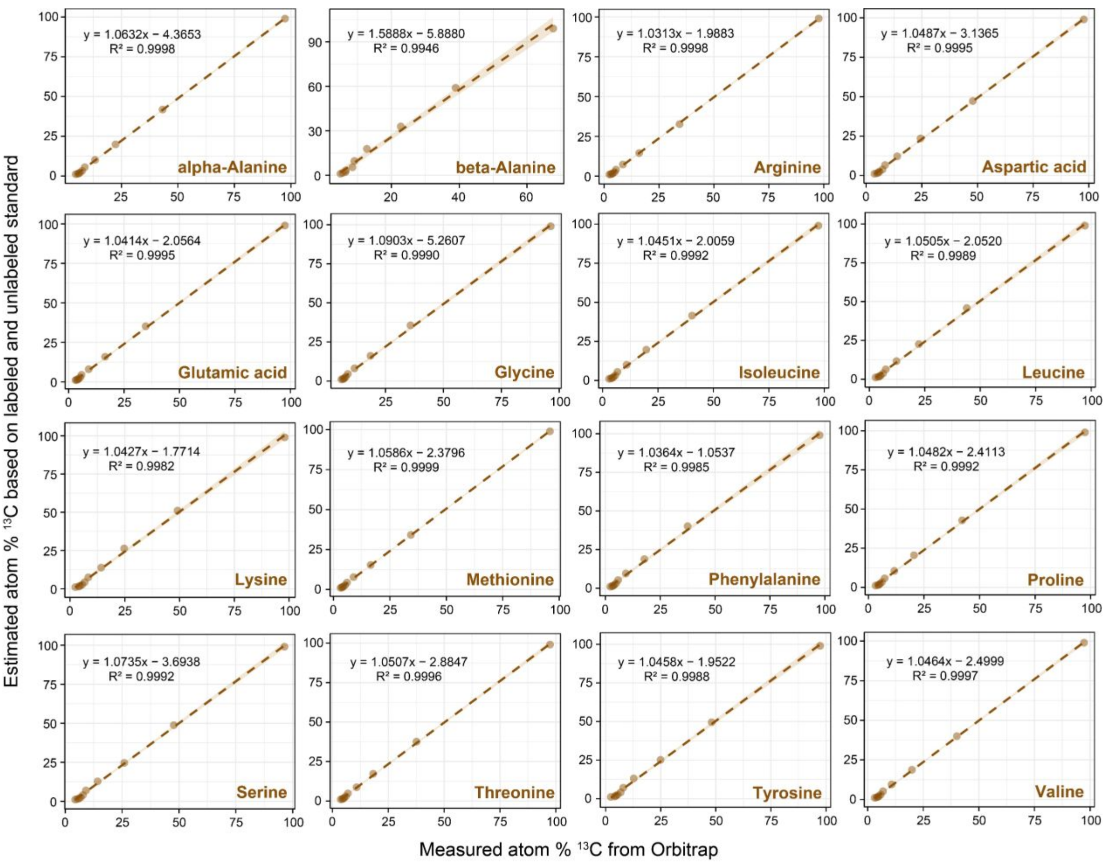

Figure S2. Isotope calibration curves of individual standard amino acids for the full isotope ranges (1.1 to 99 atom % ^{13}C) based on linear regression. Linear fitting *p* values for all amino acids were less than 0.001.

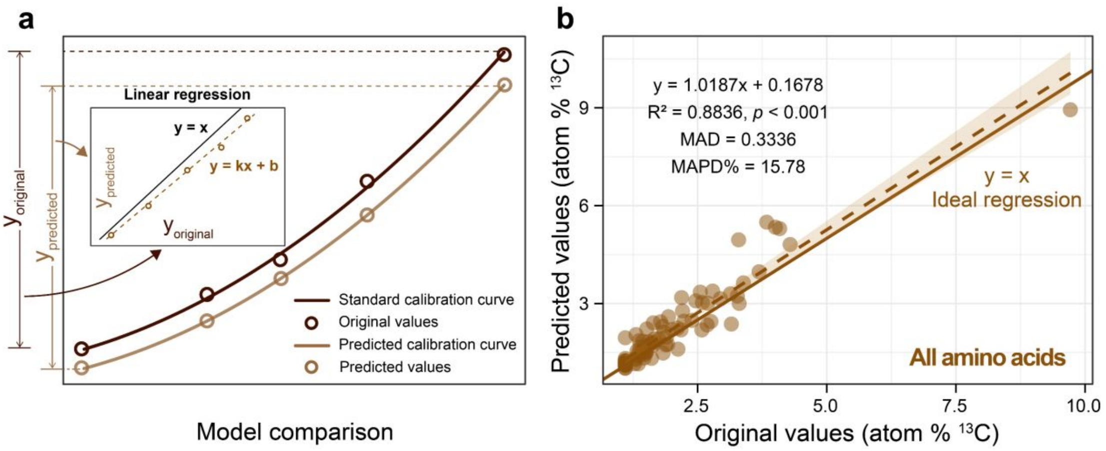

Figure S3. **(a)** Conceptual sketch of model comparison between measured (standard) calibration curves and predicted calibration curves. **(b)** Linear regression between atom % ^{13}C from original, measured isotope calibrations and predicted isotope calibrations for all amino acid standards. The solid line indicates the ideal regression line y = x, suggesting that the measured and predicted values are the same.

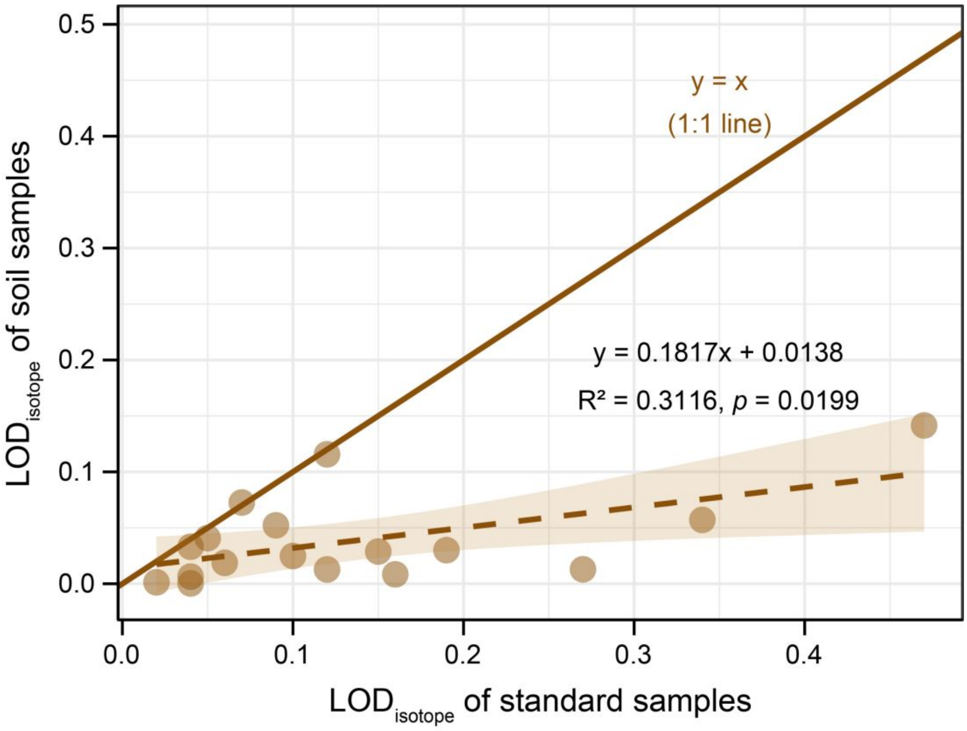

Figure S4. The relationship between the isotopic limits of detection ($LOD_{isotope}$) values of standard and soil samples to evaluate the potential matrix effect.

REFERENCES

(1) Jierula, A.; Wang, S.; Oh, T.-M.; Wang, P. Study on accuracy metrics for evaluating the predictions of damage locations in deep piles using artificial neural networks with acoustic emission data. *Appl. Sci.* **2021**, *11* (5), 2314.

(2) Uncuoglu, E.; Citakoglu, H.; Latifoglu, L.; Bayram, S.; Laman, M.; Ilkentapar, M.; Oner, A. A. Comparison of neural network, Gaussian regression, support vector machine, long short-term memory, multi-gene genetic programming, and M5 Trees methods for solving civil engineering problems. *Appl. Soft Comput.* **2022**, *129*, 109623.

(3) Chen, Z.; Zhao, M.; Lv, Y.; Wang, I.; Tariq, G.; Zhao, S.; Ahmed, S.; Dong, W.; Ji, G. Higher heating value prediction of high ash gasification-residues: comparison of white, grey, and black box models. *Energy* **2024**, *288*, 129863.

(4) Du, Z.; Sun, X.; Zheng, S.; Wang, S.; Wu, L.; An, Y.; Luo, Y. Optimal biochar selection for cadmium pollution remediation in Chinese agricultural soils via optimized machine learning. *J. Hazard. Mater.* **2024**, *476*, 135065.
